# *In-vivo* Raman microspectroscopy reveals differential nitrate concentration in different developmental zones in Arabidopsis roots

**DOI:** 10.1186/s13007-024-01302-3

**Published:** 2024-12-18

**Authors:** Alma Fernández González, Ze Tian Fang, Dipankar Sen, Brian Henrich, Yukihiro Nagashima, Alexei V. Sokolov, Sakiko Okumoto, Aart J. Verhoef

**Affiliations:** 1 Department of Soil and Crop Sciences, Texas A&M University and Texas A&M AgriLife Research, College Station, TX USA; 2 Institute of Quantum Science and Engineering, Texas A&M University, College Station, TX USA; 3 Department of Physics and Astronomy, Texas A&M University, College Station, TX USA; 4 Department of Horticultural Sciences, Texas A&M University and Texas A&M AgriLife Research, College Station, TX USA

**Keywords:** Raman spectroscopy and microscopy, Nitrate uptake and storage, Root imaging, Arabidopsis thaliana, *In-vivo* mapping, *In-situ* non-destructive measurement

## Abstract

**Background:**

Nitrate (NO_3_^−^) is one of the two major forms of inorganic nitrogen absorbed by plant roots, and the tissue nitrate concentration in roots is considered important for optimizing developmental programs. Technologies to quantify the expression levels of nitrate transporters and assimilating enzymes at the cellular level have improved drastically in the past decade. However, a technological gap remains for detecting nitrate at a high spatial resolution. Using extraction-based methods, it is challenging to reliably estimate nitrate concentration from a small volume of cells (i.e., with high spatial resolution), since targeting a small or specific group of cells is physically difficult. Alternatively, nitrate detection with microelectrodes offers subcellular resolution with high cell specificity, but this method has some limitations on cell accessibility and detection speed. Finally, optical nitrate biosensors have very good (*in-vivo*) sensitivity (below 1 mM) and cellular-level spatial resolution, but require plant transformation, limiting their applicability. In this work, we apply Raman microspectroscopy for high-dynamic range *in-vivo* mapping of nitrate in different developmental zones of Arabidopsis thaliana roots *in-situ*.

**Results:**

As a proof of concept, we have used Raman microspectroscopy for *in-vivo* mapping of nitrate content in roots of Arabidopsis seedlings grown on agar media with different nitrate concentrations. Our results revealed that the root nitrate concentration increases gradually from the meristematic zone (~ 250 µm from the root cap) to the maturation zone (~ 3 mm from the root cap) in roots grown under typical growth conditions used for Arabidopsis, a trend that has not been previously reported. This trend was observed for plants grown in agar media with different nitrate concentrations (0.5–10 mM). These results were validated through destructive measurement of nitrate concentration.

**Conclusions:**

We present a methodology based on Raman microspectroscopy for *in-vivo* label-free mapping of nitrate within small root tissue volumes in Arabidopsis. Measurements are done *in-situ* without additional sample preparation. Our measurements revealed nitrate concentration changes from lower to higher concentration from tip to mature root tissue. Accumulation of nitrate in the maturation zone tissue shows a saturation behavior. The presented Raman-based approach allows for *in-situ* non-destructive measurements of Raman-active compounds.

**Supplementary Information:**

The online version contains supplementary material available at 10.1186/s13007-024-01302-3.

## Background

Nitrogen (N) is almost always a limiting nutrient for plant growth in the natural environment. Ammonium (NH_4_^+^) and nitrate (NO_3_^–^) are the major forms of N in the soil to support plant growth. Excessive ammonium is toxic to plants [10] and thus, ammonium uptake is suppressed through transcriptional and post-transcriptional regulations limiting its accumulation [9, 25]. Nitrate, however, can be accumulated in the vacuole as storage when excess is available [20]. As such, plants have developed intricate mechanisms to sense, absorb, and store NO_3_^–^. It is well known that nitrate induces massive transcriptional modifications, some of which are independent of the downstream assimilations [5, 28, 46, 51, 57, 65, 71]. Such adaptive responses result in alternation in phenotypes such as root architectural changes, which ensures effective foraging of nitrate [3, 51, 57, 71]. Recent advances in high-resolution transcriptomics revealed that nitrate responses at the transcriptional level are often either cell- or tissue-type-specific [32, 41, 44]. Such local responses are realized by either the expressions of specific factors such as sensory modules [45], downstream response genes, or the difference in the local nitrate concentration. Still many fundamental aspects of nitrate uptake, such as which tissues are mainly responsible for nitrate uptake, whether nitrate uptake is restricted to specific developmental zones, or whether nitrate uptake is distributed all along the roots, are not well understood [17, 39, 63]. Therefore, developing new tools for mapping the nitrate concentration at a higher resolution (e.g., at a specific tissue or organelle level) will aid in achieving a better understanding of the local nitrate signals in relation to tissue/cell specific molecular mechanisms. However, it is challenging to estimate nitrate concentration reliably from a small tissue volume using extraction-based methods as small-scale extraction is physically difficult. Different methods have been used for nitrate detection in plant tissue, including nuclear magnetic resonance [7], high-performance liquid chromatography [43, 67], ion chromatography [61], radioactive isotopes [19], salicylic acid method [16, 62, 72], phenol sulfonic acid method [16], gas chromatography-mass spectrometry, gas chromatography [15], and secondary ion mass spectroscopy. Many of these techniques are destructive and lack spatial resolution, require laborious sample preparation, and/or the use of toxic reagents, and are susceptible to interferences.

Nitrate-selective microelectrodes have been used for intracellular measurements of nitrate activities/concentration in excised barley tissue, leaves and vacuolar cells of barley roots [48, 49, 61, 64]. Microelectrode methods are reported to be more appropriate for cells at the surface of tissues. Accessing cells beyond the surface layer causes issues because of damage to cells that are above the cell of interest, as well as associated artifacts and complications in the interpretation of the measured potentials [48]. Optical imaging methods based on fluorescent biosensors offer an alternative to probe biological tissue with high spatial and temporal resolution since light can be used for probing living systems non-destructively. Nitrate sensing with fluorescence microscopy has been achieved with genetically encoded fluorescent nitrate sensors based on nitrate transporters or nitrate-binding-domain proteins, both of which are endogenous nitrate sensors [17, 18, 27, 36]. The transporter-based sensors detect transporter’s activities, which in turn could imply the flow of nitrate. However, the signals only reflect changes to the conformation of the transporters, which might not be associated with the local nitrate concentration. Nitrate sensors, like mCitrine-NLP7, have been used to estimate cytosolic nitrate concentrations in Arabidopsis. More recently a nuclear-localized genetically encoded fluorescent biosensor [17] was used for *in-vivo* visualization of nitrate distribution and dynamics in Arabidopsis thaliana. While having advantages such as excellent spatial resolution and non-destructive measurements, this type of sensors typically has a detection range of ~ 2 orders of magnitudes around the dissociation constant [52]. As a consequence, the fluorescence response of this sensor to nitrate shows saturation starting below 1 mM, and mCitrine-NLP7 (kd ~ 90 μM) is likely not suited for measuring concentrations above 1 mM. Moreover, these genetically encoded biosensors require plant transformation [37] which greatly limits the range of plant species that can be studied.

Raman spectroscopy is an optical technique that allows for nondestructive label-free chemical-specific identification of compounds within biological samples without requiring complex sample preparation or plant transformation. It is based on the Raman effect, an optical phenomenon in which photons are inelastically scattered by matter. Incident photons lose (or gain) energy after interacting with the sample. Most commonly this involves vibrational (or rotational) energy being gained by a molecule, while the incident photon frequency undergoes a red-shift (energy loss). The associated energy shifts can be recorded and used to obtain information about structural and molecular composition from the sample [50, 66]. In contrast to its high infrared absorption, water has only weak Raman scattering properties; therefore, Raman spectroscopy is well suited to be used with aqueous samples, and to perform studies of biological materials, including plants [31, 68]. This technique can be combined with microscopy implementations for achieving nondestructive, chemical specific *in-vivo* imaging with high spatial resolution. As the Raman effect does not require labeling, this technique avoids concerns for cytotoxicity or perturbation of sample properties induced by exogenous labeling and is naturally compatible with *in-vivo* measurements.

In plant research Raman spectroscopy has proven to be useful for the identification of chemical compounds [12, 69], and has shown great potential for the determination of pigments such as lycopene and carotene in tomatoes [5, 6], carotenoid concentrations in fruits and vegetables [8], to detect and identify drought [1] and pathogen stress [26, 47]. Nitrate status in Arabidopsis leaves was measured with a portable Raman device by measuring a characteristic nitrate peak at 1046 cm^−1^ [34]. The combination of microscopic imaging techniques with Raman spectroscopy has provided a non-invasive tool for improving spatial resolution and chemical sensitivity in tissues [2, 30, 53, 56, 70]. Raman microspectroscopy was used to detect nitrate concentrations as low as 15 mM at the adaxial leaf surface in tomato after foliar application of CaNO_3_ (Solanum lycopersicum) [13]. Confocal Raman microspectroscopy was used in a multimodal correlative approach to look into the spatial organization of roots, minerals and bacteria within the rhizosphere of maize, for which sample preparation was done using a specially designed soil resin-embedding technique [4]. Raman microspectroscopy has recently been used for *ex-vivo* detection of deuterium in excised-root cross-sections of hydroponically grown and deuterium-labeled maize [21].

In this work, we present a methodology based on Raman microspectroscopy for label-free *in-vivo* non-invasive detection of nitrate in roots of *Arabidopsis thaliana* seedlings. The measurements are done *in-situ* in intact roots of Arabidopsis seedlings grown on agar pads in petri-dishes with different nitrate concentrations. For these measurements, we use a custom-developed Raman microscopy system, which was built with readily available off-the-shelf components. Nitrate concentrations, down to a detection limit of 0.5 mM were measured with ~ 100 µm resolution along the seedling roots. The sizes of Arabidopsis root cells (especially the length) vary in the different developmental zones, from a few micrometers length in the meristematic zone to hundreds of micrometers length in the maturation zone [14], hence our experiment does not capture nitrate concentrations at the single cell level, but visualizes the volumetric spatial distribution of nitrate at an intermediate scale with tens of micrometer resolution. This resolution is well suited for acquiring nitrate concentration data within the different developmental zones encountered along the root length. Our results show that the nitrate concentration increased along the root, from the tip towards the maturation zone with the meristematic zone having the smallest concentration and the maturation zone having the highest concentration of nitrate. This work also shows a saturation behavior of nitrate concentration in the maturation zone with increasing nitrate concentration in the surrounding medium. The methodology presented in this paper shows the potential of Raman-based methods as label-free, noninvasive alternatives to study nitrate uptake in plant roots under physiologically relevant conditions and opens up alternative avenues to study and relate physiological quantities such as nutrient storage with local transcriptomics.

## Material and methods

### Seedling growth condition

Sterilized Col-0 seeds were sowed onto plates containing 0.8% agar 1 × Murashige & Skoog Modified Basal Salt Mixture without nitrogen (PhytoTechnology Laboratories™) media. KNO_3_ was supplemented as the only source of nitrogen in the following concentrations (0.5, 1, 2, 5, 10, 25, 50 and 100 mM). The plates were wrapped with parafilm® and placed standing vertically in the growth chamber set to 23 °C, with a light and dark cycle of 16 and 8 h. The nitrate concentration in the roots was measured after 5 days. For the Raman measurements, we selected (via visual inspection) seedlings that were similar in length and were phenotypically similar. For this, many seedlings (approximately 14) were grown per plate and typically 4 seedlings from each plate were used for Raman measurements. For reliable Raman measurements, it is important that roots grow on the surface of the growth medium. Roots that penetrated the agar during growth were not measured.

### Raman measurements

**Fig. 1:**
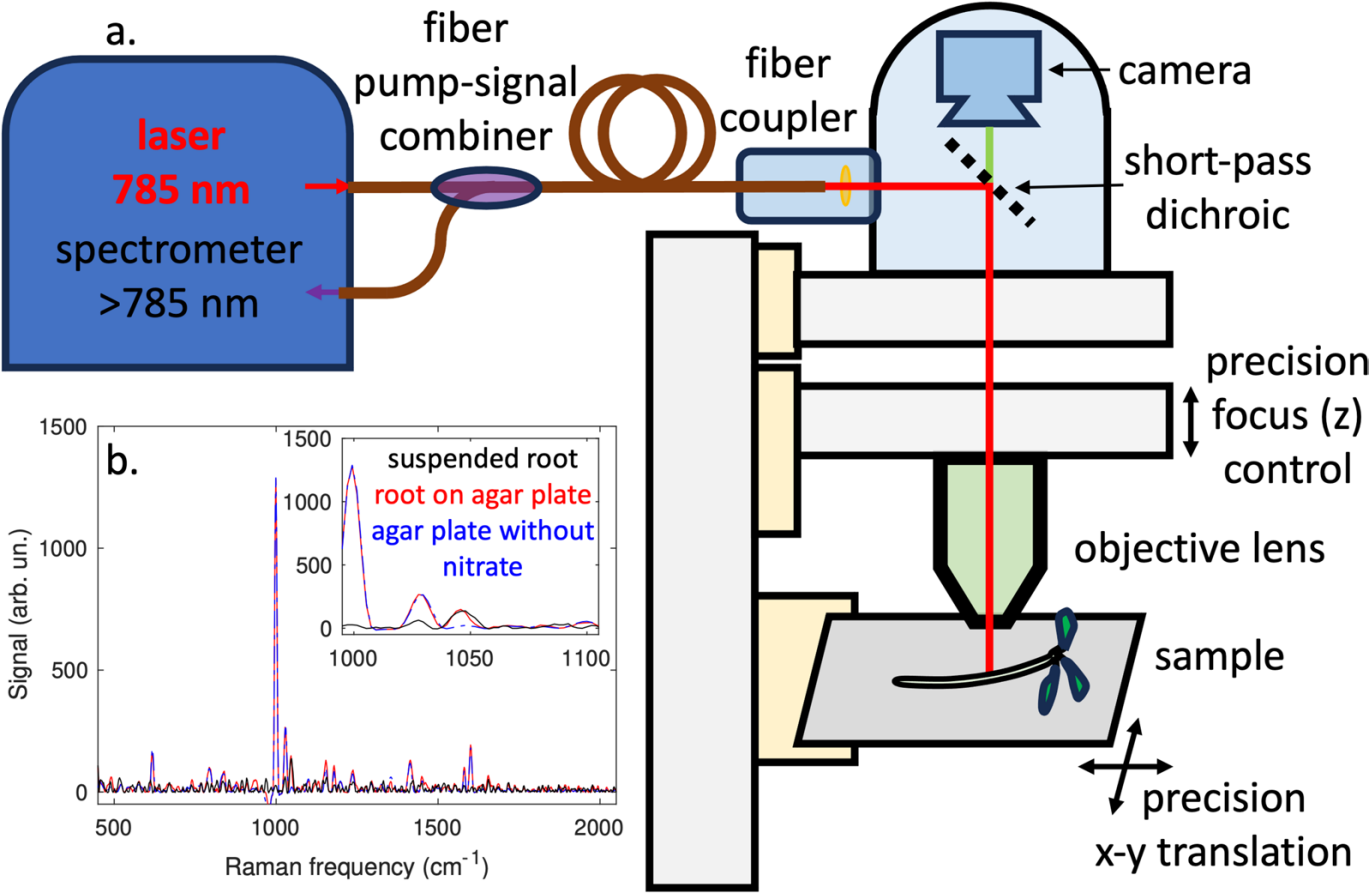
a. Artistic illustration of the physical setup of the Raman associated microscope (details described in material and methods). b. shows the Raman spectra of samples measured with root suspended in air (black solid line), root on agar plate (red solid line), or agar plate without KNO_3_ (blue dashed line)

Raman spectra were acquired using a custom-assembled microscope system by focusing the 785-nm laser light emitted by the iRaman Plus spectrometer (BWTek, Plainsboro, NJ, USA) on the roots of 5-day-old seedlings at specified distances from the root tip using a 20X long working distance objective with high infrared transmission. Using this microscope objective, the laser beam was focused to a spot diameter slightly smaller than 100 µm. The standard probe of the spectrometer was replaced by a microscope adapter, which connects to our custom-built microscope. The microscope adapter includes a short-pass dichroic beam-splitter that allows visible light to be used to inspect the roots with a camera and to precisely position the laser focus on the root, with the use of high-precision motorized sample translation stages (PLS-XY, Thorlabs Inc, Newton, NJ, USA) and focus adjustment (ZFM2030, Thorlabs Inc). With these stages the sample and focus position can be adjusted with 1-µm precision. A schematic of the system is shown in Fig. 1a. For the Raman measurements of roots, the agar plates were placed horizontally on the sample stage (Fig. 1a). To obtain the Raman spectrum, the laser light was precisely focused onto the surface of the root. The light scattered from the root tissue that goes back through the microscope objective is separated from the laser light using a short-pass dichroic beam-splitter in the spectrometer, which lets laser light (785 nm) go through but reflects off the Raman signal. The Raman signal is collected via a fiber-bundle and directed to the Raman spectrometer where the Raman signal is recorded as a function of the wavelength shift. BWSpec 4.10 software supplied with the spectrometer was used for data acquisition. For nitrate detection, we used the peak observed at ~ 1046 cm^−1^ associated with the symmetrical stretching of nitrate molecules, [38, 40]. For this work, we measured Raman spectra from roots grown in agar plates with different nitrate concentrations. Raman measurements were done in-situ, i.e. without removing the seedlings from the agar medium. The acquisition parameters (integration/exposure time, laser power and number of averaged spectra) were determined in a set of preliminary Raman measurements in roots and agar plates and were chosen in such a way that enough signal-to-noise ratio of the data for the seedlings grown in the lowest used nitrate concentration (0.5 mM) could be obtained while preserving the sample integrity. Spectra were acquired with the laser power set to 30% (resulting in 98 mW measured after the 20X microscope objective), a laser exposure time of 30 s and 10 averaged spectra at each measurement point. The same set of parameters was used for all Raman measurements presented in this work.

Once the set of acquisition parameters was determined we performed systematic measurements of Raman nitrate detection, first in agar plates with different nitrate concentrations (0–100 mM). For these measurements, the laser beam was precisely focused on the surface of the agar pad and spectra were taken across the agar plates (up to 50 different positions were measured per plate). Nitrate concentration measurements in roots were done for roots grown in on agar pads with different nitrate concentrations (0.5 mM, 1 mM, 2 mM, 5 mM, 10 mM). For each root, Raman spectra were taken at seven different locations along the root. These points were located at 250 µm, 500 µm, 1 mm, 2 mm, 4 mm, 6 mm and 8 mm distance from the root tip.

Given the 30 s laser exposure time and the ten averages per sampled location, the Raman spectrum acquisition time was 5 min per data point. Statistical analysis was done using SAS® PROC GLIMMIX, by plate concentration. Pearson correlation was calculated using Microsoft-Excel.

### Raman data analysis

The obtained raw spectra contain not just the desired Raman signal but also background (e.g. fluorescence from the sample and spectrometer dark counts) and noise that need to be removed from the recorded spectra. Before each set of measurements was performed, a dark spectrum was taken. This spectrum is obtained by using the same integration time and number of averages, but the 785 nm laser used to illuminate the sample was turned off. The BWSpec 4.10 automatically subtracts this dark spectrum from each measured spectrum. For background removal (baseline correction) [24, 33, 35] and spectral smoothing, we used built-in algorithms of the BWSpec 4.10 spectroscopy software (also used for data acquisition). For baseline subtraction, the manufacturer specifies that a least-squares fit of a polynomial is calculated, and subsequently subtracted from the spectrum. For spectral smoothing, we used Savitzky-Golay smoothing [58] with a window size setting of 3. A calibration curve to estimate nitrate concentration from Raman spectra was obtained by plotting the peak height of the nitrate peak at ~ 1046 cm^−1^ as a function of the nitrate concentration in agar plates (with different nitrate concentrations and without seedlings), as shown in Supplementary Fig. S1.

### *Ex-vivo* root segment nitrate concentration estimation

Nitrate concentration in root segments was estimated using an adapted version of the salicylic acid protocol reported in [72]. Since pulling roots out from the agar medium can easily damage the roots, to minimize this risk, we used an additional layer of cellophane between the media and the seeds. Growing conditions are otherwise the same as described above. From each replication, to have enough root material for reliable measurements, one hundred individual seedling roots were measured, and two 3 mm sections were harvested per root. One 3 mm section was measured starting from the root tip (0–3 mm), and the next 3 mm section (3–6 mm) was measured and taken as the second section. 100 sections of 3 mm from each distance (0–3 mm and 3–6 mm) were gathered and put in 30 µl of distilled water in separate tubes, then stored in liquid nitrogen and homogenized using metal beads. A 10 µl aliquot was used for the nitrate content measurement (following a modified procedure adopted from [72]). The root extract aliquot was combined with 40 µl salicylic acid-sulfuric acid mix (5% w/v), then neutralized using 950 µl 8% (w/v) NaOH and measured at OD_410_. Lastly, the nitrate concentrations were calculated from a KNO_3_ standard curve (produced using the same protocol). Statistical significance was determined using ANOVA and TukeyHSD in R.

### Root volume estimate

Images from 5-day-old roots were analyzed using ImageJ. Initially, 0–3 mm and 3–6 mm sections were measured from the tip in each image representing the length (L), then the perimeters of each root segment were traced, and the internal areas (A) were calculated. The area was then used to solve for the average root width (W) (using the approximation W = A/L). Lastly, assuming the root as a perfect right cylinder, average root width was used as diameter (d) to estimate the root volume (V) (using height (h) = L; $V= \pi h{(\frac{d}{2})}^{2}$). Statistical significance was determined using ANOVA and TukeyHSD in R.

### Root zone estimate

After 5 days, the seedlings were harvested and incubated in propidium iodide (10 µg/ml) for 10 min to visualize the cell wall. The images were obtained with a Nikon D-ECLIPSE C1si confocal laser scanning microscope (× 60 Plan Apo objective lens, excitation 561 nm, emission 605 nm). The images were processed by NIS-Elements AR (Nikon). Root zones were estimated based on cell elongation characteristics described in [55].

## Results and discussion

### Nitrate concentration measurements

For mapping nitrate concentration along the roots of wild-type Arabidopsis seedlings grown in agar pads, we used the nitrate peak detected at 1046 cm^−1^ [38]. A calibration curve for the peak height as a function of the nitrate concentration in agar was obtained first as described above. The height of the 1046 cm^−1^ nitrate peak in this calibration measurement showed a linear dependence on the nitrate concentration with an R^2^ coefficient of 0.9998 (Supplementary Fig. S1). In order to verify the homogeneity of the nitrate concentration on the agar plates, spectra were taken at up to 50 different positions throughout the plates, and no significant inhomogeneities were detected. The lowest concentration of nitrate that can be detected with our system above the noise level was around 0.5 mM. Analysis of the Raman spectra obtained in agar indicates we can use the height of the nitrate peak in Raman measurements to obtain information on nitrate concentrations, providing fixed acquisition parameters and assuming losses (such as absorption and scattering) are the same between different measurements (sampled points). The reliability of using our Raman microscopy system for detecting nitrate in agar and accurately measuring nitrate concentration in agar is shown by the high R^2^ value obtained. For our *in-situ* Raman measurements in roots, it is important to cross-check that our *in-situ* Raman measurements do not have contributions of the nitrate in the agar pad. For this, we compared the 1046 cm^−1^ peak height of roots measured *in-situ* on the agar pads to the peak height obtained from the same roots taken off the agar pad. For the seedlings that were removed from the agar pad, the roots were laid on a special 3D-printed microscope slide with 1-mm wide gaps so that the beam would only interact with the roots. These measurements yielded the same values (see Fig. 1b) of nitrate peak height for the measurements of roots *in-situ* and for the measurements of roots off the agar medium. This result indicates that the *in-situ* Raman measurements reflect the nitrate content in the roots without interference from the growth medium. These measurements also show the reproducibility of the Raman results in consecutive measurements. *In-situ* measurements of the roots on the agar pad are very important for studies that aim to monitor changes in root concentration over time. In addition to that, *in-situ* measurements are preferable since 5-day-old Arabidopsis roots are fragile and can be damaged when pulling them off the agar plate and mounting them properly on our 3D-printed slide for imaging. Furthermore, the seedling roots tend to dry up outside the agar causing the roots to shrink and change shape.

**Fig. 2:**
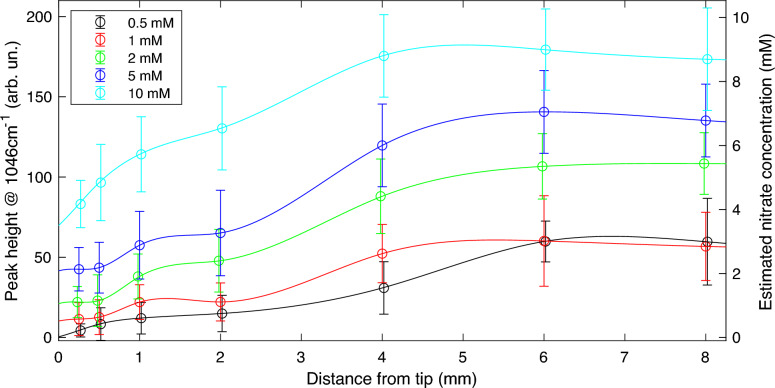
Graph showing the Raman signal corresponding to nitrate (left Y-axis) and corresponding concentration estimate (right Y-axis) for root samples measured from 0.5 (black), 1 (red), 2 (green), 5 (blue), and 10 (cyan) mM [KNO_3_] plates at various distances from the tip (i.e., on the X-axis, 0.25, 0.5, 1, 2, 4, 6 and 8 mm). At 10 mM and 1 mM, 30 roots were measured, at 5 mM 33 roots, at 2 mM 32 roots, and at 0.5 mM 9 roots. The open circles represent the average of measured data points, error bars are defined as the standard deviation of all the measured values at a given position and agar pad KNO_3_ concentration. The solid lines are cubic splines fitted through the measured data points for visualization of the observed trends

Next, we investigated how the nitrate concentration along the root(s) changes under different nitrate concentrations in the agar pad. Spatial variation of the nitrate concentration in roots grown on plates with different nitrate concentrations (i.e., 0.5, 1.0, 2.0, 5.0 and 10.0 mM) was measured by taking Raman spectra at seven positions along the root (at distances of 0.25, 0.5, 1.0, 2.0, 4.0, 6.0 and 8.0 mm from the root tip). Macroscopically, the seedlings grown in agar plates with nitrate concentrations between 0.5 to 10 mM were phenotypically similar (Supplementary Fig. S2). For seedlings grown in agar plates with high nitrate concentrations (25, 50, and 100 mM), a retardation in root growth was observed, hence seedlings grown in agar with nitrate concentrations higher than 10 mM were not included in further studies. Root hair development was found to be retarded at higher concentrations (i.e., 10 mM) versus lower ones (e.g., 1 mM) as has also been observed in other studies [60]. The nitrate peak height obtained from the Raman measurements in roots was plotted as a function of the distance from the tip, as shown in Fig. 2. We used the calibration curve obtained from the measurements of nitrate peak height versus nitrate concentration in agar to estimate nitrate concentrations in roots. These results are shown with the scale on the right Y-axis in Fig. 2. The data shows a strong positive correlation (R^2^ = 0.96) between media concentration and (averaged) Raman measurements from the root tissues (Supplementary Fig. S3), showing that the nitrate uptake capacity of Arabidopsis roots is not saturated up to an agar nitrate concentration of 10 mM. We observed a significantly lower nitrate concentration (Fig. 2 and Supplementary Table S1) in the younger cells of the root (i.e., 0.25, 0.5, 1.0, and 2.0 mm from the root tip) compared to the older section (i.e., 4.0, 6.0 and 8.0 mm from the root tip). This trend was observed for roots grown in all different plate concentrations studied in this work (0.5 mM to 10 mM). To our knowledge this phenomenon has not been reported in any previous work.

This remarkable result is obtained despite the practical challenges of working with plant roots. Root tissue has a complex structure, with a different morphology and composition compared to the agar medium samples. Hence, Raman measurement conditions, like optical aberrations, scattering, signal background, sample composition, are different in root tissue with respect to the calibration curve obtained from agar pad samples. Consequently, for the same laser excitation conditions, the average excitation intensity and signal collection efficiency are different (lower) and this makes exact concentration measurements from biological samples challenging. Nevertheless, since all acquisition parameters across our measurements were kept constant, relative comparisons between comparable samples can provide very useful information about nitrate concentration profiles and nitrate accumulation differences along the root.

### Verification (cross-validation) of Raman measurements

As discussed in the section on “*Ex-vivo* root segment nitrate concentration estimation”, to confirm the observations presented in Fig. 2, we independently measured the nitrate concentration in two sections from 5-day old seedling roots (0–3 mm and 3–6 mm, which we refer to as tip and mature sections, respectively). This was done using an adapted version of the salicylic acid protocol reported in [72]. External nitrate concentrations (nitrate concentration in the agar pad) of 5 mM and 10 mM were chosen because of the robust nitrate concentration gradient observed from the tip to the maturation zone under these concentrations (Fig. 2). The 3 mm increment was chosen because this length permitted highly confident measured sectioning of roots under a dissection microscope and allowed collecting sufficient root material from 100 seedling roots to perform reliable nitrate quantification. Each 100 root sections from either the tip sections or mature sections constituted as a single sample used for the salicylic acid nitrate measurement assay.

**Fig. 3:**
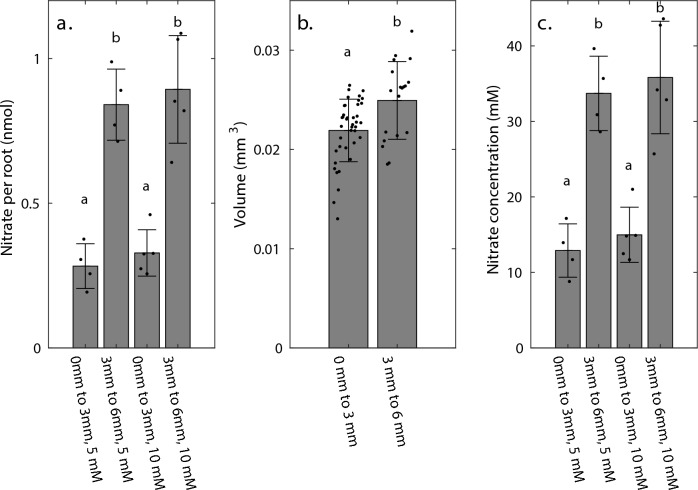
a. Bar graph showing the estimated nitrate amount (from salicylic acid method, nmol) of root sections collected from 0–3 mm and 3–6 mm of roots grown on 5 mM and 10 mM [KNO_3_] plates. b. Bar graph showing the estimated root volume (mm^3^) estimated based on root images (0–3 mm, n = 40; 3–6 mm n = 19). c. Bar graph showing the normalized concentration of nitrate per root of 0–3 mm and 3–6 mm section grown on 5 mM and 10 mM [KNO_3_] plates

As shown in Fig. 3a, the measured nitrate content for the tip-sections was 0.28 and 0.33 nmol per root section for the roots grown in 5 mM and 10 mM respectively; for the mature sections, the nitrate content was 0.84 and 0.89 nmol, respectively. The volume estimates were 0.022 and 0.025 mm^3^ for the tip and mature sections, respectively (Fig. 3b). Using the nano-molar nitrate measurements (Fig. 3a) and volume estimates (Fig. 3b), the nitrate concentration is calculated as the values shown in Fig. 3c. For the 5 mM plate, the tip and mature sections had average concentrations of 12.9 and 33.7 mM, while for the 10 mM plate, the average concentrations were 15.0 and 35.8 mM, respectively. Statistically significant differences are shown as letters in each panel. Interestingly, the tip sections had a statistically lower nitrate concentration compared to the mature root sections (i.e., alpha = 0.05, 5 mM plate Adj. P = 0.0011, 10 mM plate Adj. P = 0.0001), while no significant difference was detected when comparing the same sections from the 5 mM and 10 mM plates (e.g., 0–3 mm sections from 5 mM vs. 0–3 mm sections from 10 mM).

It is worth noting that our validation measurement through destructive sampling required 100 root sections of 3 mm length per sample, which is extremely time-consuming to harvest even at this resolution. If destructive sampling were to be conducted at 100 µm resolution to match our Raman measurements, thousands of sections would be required, making such an experiment highly impractical.

For seedlings grown in agar with nitrate concentrations of 5 mM and 10 mM, the (weighted average) Raman estimates in the 0–3 mm section ranged between 2.7 mM and 6 mM, and in the 3–6 mm section between 6.2 mM and 9.05 mM, see Supplementary Table S2. Nitrate concentration measurements using the salicylic acid method yielded higher values, but also reveal a higher nitrate content for the more mature roots section (3–6 mm) as compared to the younger section of the root (0–3 mm). In fact, the nitrate concentrations estimated using Raman were on average about four times lower. This difference in absolute values measured with these two methods is to be expected since for the salicylic acid method the nitrate contained in the whole root section is extracted (after destroying the cell walls and membranes), while for Raman microscopy, the contributions of different parts of the roots along the beam propagation direction is not equal. This is because of absorption and scattering (which is expected to be especially strong due to cell walls in plant tissues), change the intensity of the excitation light across the root, which makes the generated and detected Raman signal dependent on depth from the surface of the root. The calibration curve used to estimate the nitrate concentration from the Raman measurements was obtained from agar containing nitrate, which has optical properties different from root tissue. Given the expected stronger scattering in the root tissue, the nitrate concentration values calculated for the root are expected to underestimate the actual nitrate concentration. Thus, Raman microscopy can be utilized for comparing relative concentrations reliably among tissues with similar optical properties, but not to provide absolute values of nitrate concentrations based on the calibration curve obtained from nitrate containing agar plates. In Supplementary Table S2, we show the results of averaging the nitrate concentration values, using the interpolated values obtained from the cubic spline curves used for fitting the nitrate concentration vs. distance from the tip. For roots grown on agar pads with 5 mM nitrate, the average nitrate concentration estimated by Raman in the 3–6 mm section are found to be 2.0 times higher compared to the 0–3 mm section. This ratio is 1.5 for roots grown on agar pads with 10 mM nitrate. The ratios found with the destructive colorimetric measurement were 2.6 and 2.4, respectively. When calculating concentration values from the Raman measurements for the root tip and more matured root sections, without including the interpolated concentration values for distances encompassed between 2 and 4 mm measured from the tip (where no Raman measurements were done in the roots), the ratios from the Raman measurements yield 2.5 and 1.7, respectively. Therefore, both methods show a higher concentration of nitrate in the more matured zone of the roots.

**Fig. 4.**
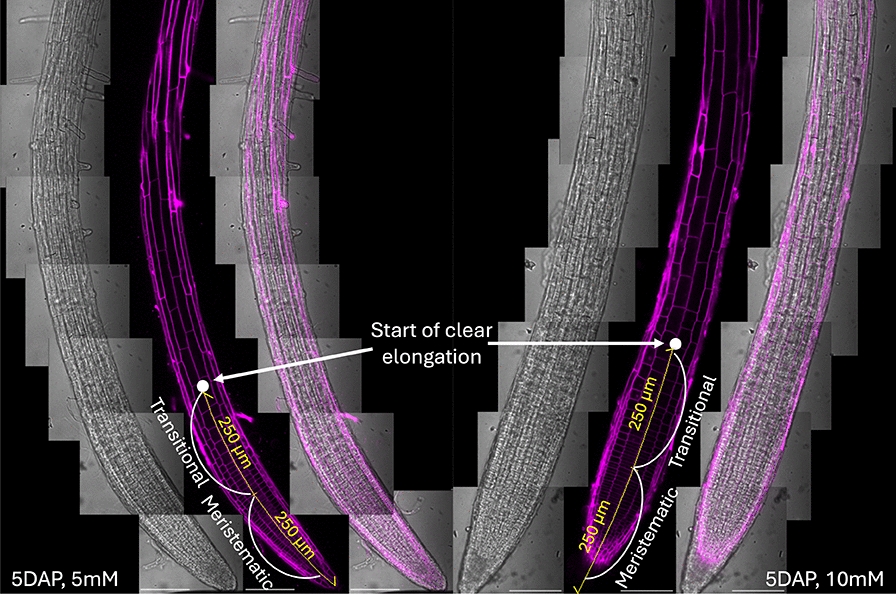
5 days after planting (DAP), PI stained-root images from 5 and 10 mM [KNO_3_] plates. White dots indicate the starting point of clear root elongation. Root zones (indicated by arched white line) are marked in yellow text with 250 µm increments (in yellow line) measured from root tip

The observation of the root tip having consistently lower nitrate concentration is likely due to the different developmental zone characteristics. No obvious developmental differences were observed between roots grown on 5 mM and 10 mM plates (Fig. 4, additional roots shown in Supplementary Fig. S4). The first and second spots of the Raman readings are within the meristematic (i.e., ~ 250 µm from root cap) and elongation (i.e., beyond ~ 500 µm from root cap) zones, respectively, and both zones are characterized by having an absence or immature vasculature system [55]. Fitting to this observation, mining previously published high-resolution transcriptome data revealed that the expression level of low-affinity nitrate transporters (NPF6.3/NRT1.1 and NPF4.6/NRT1.2) is lower in the meristematic zone, indicating that the nitrate uptake capacity of this zone might be lower than the elongation and maturation zone [23] (Supplementary Table S3). Furthermore, as described in depth by [42], vacuole maturation and expansion happens rapidly in the root elongation zone. The expression of vacuolar nitrate transporters (CLCa and CLCb) seems to reflect this difference, also being lower in the meristematic zone (Supplementary Table S3). The expanded vacuole can take up ~ 90% of the space inside of a cell, which provides the driving force for cell elongation due to its salt accumulation capacity (i.e., high osmotic potential) [11]. Vacuoles are known to accumulate nitrate at a higher concentration due to concentrative transport [22, 29]. Hence the observed nitrate distribution in the root could be a result of increased uptake and vacuolar storage capacity in the mature root. Modeling flux and metabolism based on high-resolution transcriptomics and proteomics, combined with the genetic resources in Arabidopsis and Raman microscopy, will permit to evaluate the source of such a concentration gradient.

## Conclusion

In this study, we present a methodology for *in-situ in-vivo* non-destructive mapping of nitrate in *Arabidopsis thaliana* roots based on Raman microspectroscopy. A calibration model to accurately predict the nitrate concentration in the agar medium was obtained. A comparison of the nitrate concentration obtained using the calibration model and obtained with wet chemistry indicates that the calibration model underestimates the actual nitrate concentration in roots. This discrepancy was to be expected, given that the optical properties of the agar medium and the root tissue are different. Further studies may allow to develop better calibration models, such as accurate root tissue phantoms, in order to provide accurate nitrate concentration values in root tissue using Raman spectroscopy. Since the presented methodology used the same acquisition parameters for all the measurements, comparisons of relative nitrate concentration can be reliably obtained. The possibility of measuring the roots without removing the seedlings from the growth medium is advantageous for studies that require monitoring nitrate concentration changes over time. The per-point acquisition time of 5 min, used in this work, was required to obtain a signal-to-noise ratio sufficient for measuring nitrate in media with low nitrate concentration (0.5 mM). However, for roots grown in higher nitrate concentration (> 2 mM), single shot measurements can be reliably done with the presented system, which would be helpful for studies that require many more roots to be measured, or many repeated measurements of the same root, for example to study changes over time. The advantage of ease of usage as compared to other molecular techniques enabled speedy and reliable (relative) nitrate estimations in live tissues. Our results show that nitrate concentration increases along the root developmental zones, with higher concentration of nitrate measured in the maturation zone and the lowest nitrate concentration in the meristematic zone. Our study also shows a saturation behavior for nitrate accumulation in the maturation zone. Further studies are required to conclusively explain the observed nitrate concentration gradient along the roots. These results show potential for using Raman-based approaches with higher spatial and temporal resolution, such as confocal Raman microscopy and coherent Raman techniques [2, 59] for sub-cellular mapping of nitrate concentration. Although both techniques allow for micrometer-resolution measurements, implementations using coherent Raman will be advantageous for increasing detection speed and for in-depth high-resolution measurements [54]. The label-free nature of Raman spectroscopy has the advantage of not requiring plant transformation. The presented methodology can be extended to study other types of plants, and could be of interest to breeding crops with higher nitrate uptake capability.

## Supplementary Information

Supplementary Material 1

## Data Availability

The datasets used and/or analysed during the current study are available from the corresponding author on reasonable request.
