# Supplementary material for: In-vivo Raman microspectroscopy reveals differential nitrate concentration in different developmental zones in Arabidopsis roots
Source: Plant Methods. 2024 Dec 18;20:185. doi: 10.1186/s13007-024-01302-3 (PMC11657419; doi:10.1186/s13007-024-01302-3)
Supplement: Supplementary file 1 — Supplementary Material 1 [file 13007_2024_1302_MOESM1_ESM.pdf]

Supplementary Figures and Tables

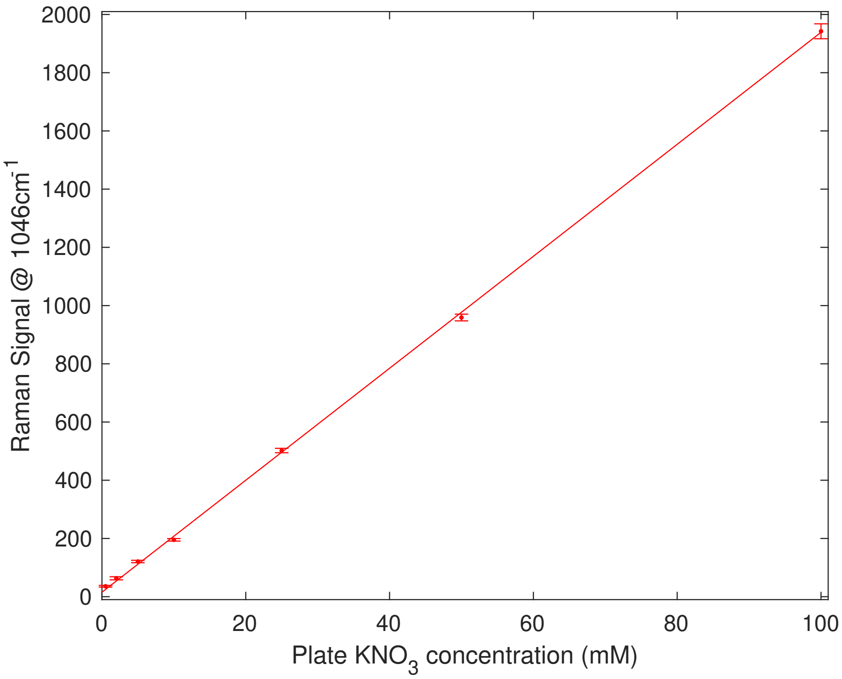

Supplementary Figure S1. Average Raman nitrate peak signal value when focused on the agar pad (Y-axis) versus KNO_3 concentration in agar pads (X-axis). Error bars are defined as the standard deviation of all measured values per KNO_3 concentration, divided by the square-root of the number of measurements at each concentration. The fit yields a slope of 19.23±0.28 and intercept of 15.36±19. The R^2 value of the fit is 0.9998.

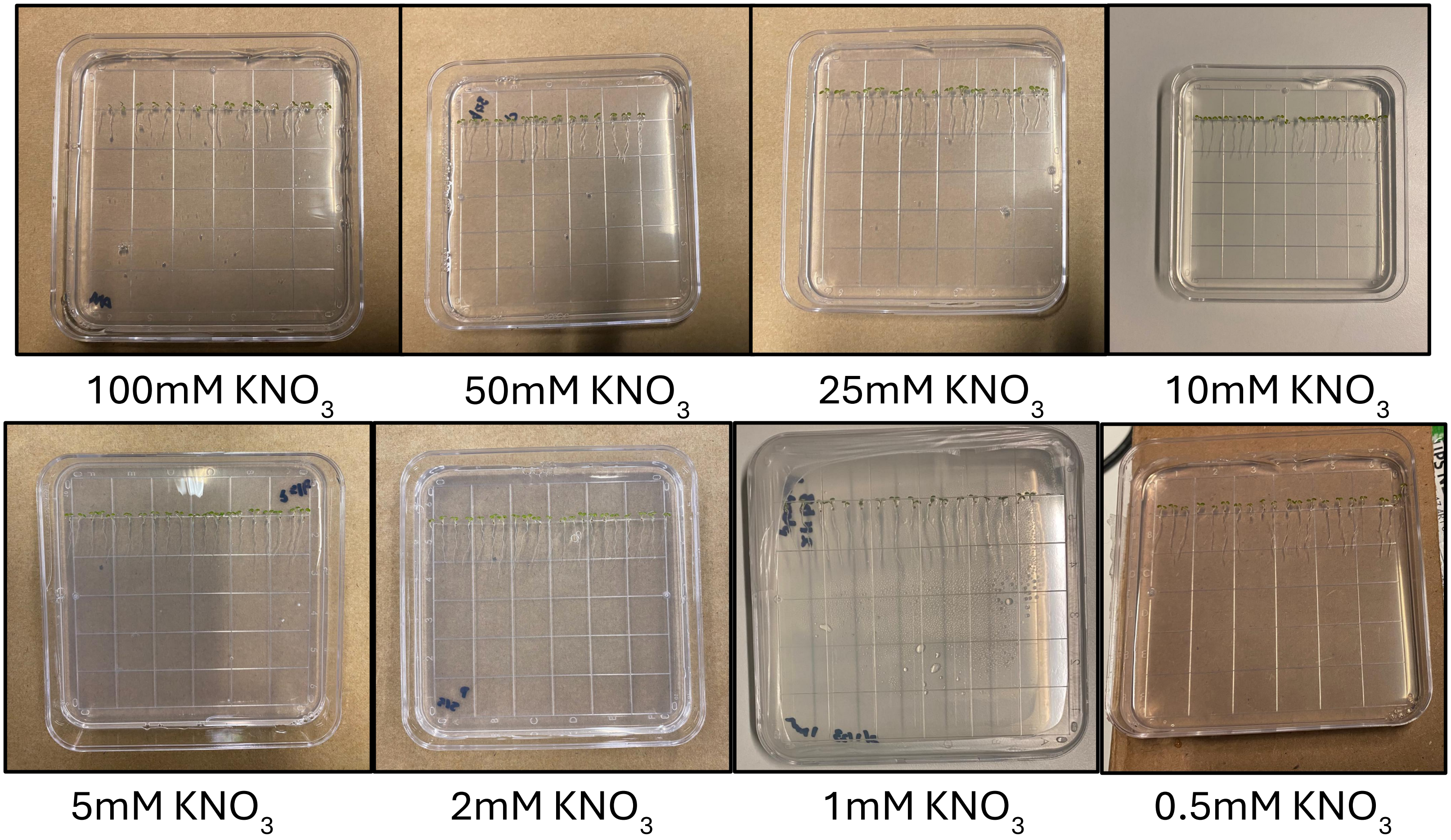

Supplementary Figure S2. Root phenotype grown on various concentrations of KNO_3 MS media. For scale note that the grid pattern on the bottom of each plate forms 0.5 inch squares.

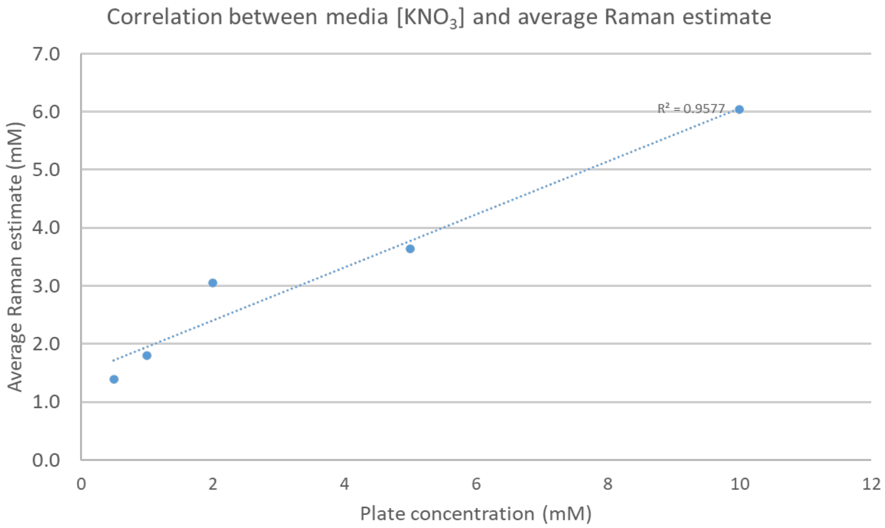

Supplementary Figure S3. Correlation graph between plates nitrate concentration (X-axis) versus average Raman nitrate estimate from roots grown on each corresponding plate. The calculated average does not take into account that the measurement points are not evenly spaced, causing the measured concentration closer to the root tip to be weighed more strongly than further away from the root tip.

Distance (mm)	0.5 mM	1.0 mM	2.0 mM	5.0 mM	10.0 mM
0.25	A	A	A	AB	A
0.5	A	A	A	A	A
1	A	A	B	B	AB
2	A	A	B	B	B
4	B	B	C	C	C
6	C	C	D	D	D
8	C	BC	D	D	CD

Supplementary Table S1. A table showing the pair-wise significance (Conducted in SAS® using PROC GLIMMIX, analyzed by each plate nitrate concentration) of each measured distance (first column).

Plate [KNO_3] (mM)	Average interpolated Raman (mM)			
	0-3 mm	3-6 mm	0-2 mm	4-6 mm
0.5	0.64	1.97	0.52	2.32
1	1.07	2.69	0.94	2.95
2	2.10	4.63	1.77	5.02
5	3.07	6.23	2.73	6.74
10	5.99	8.80	5.45	9.05

Supplementary Table S2. The averaged interpolated Raman estimates of nitrate concentration from 0-3 mm, 3-6 mm, 0-2 mm and 4.6 mm collected from each [KNO_3] plates.

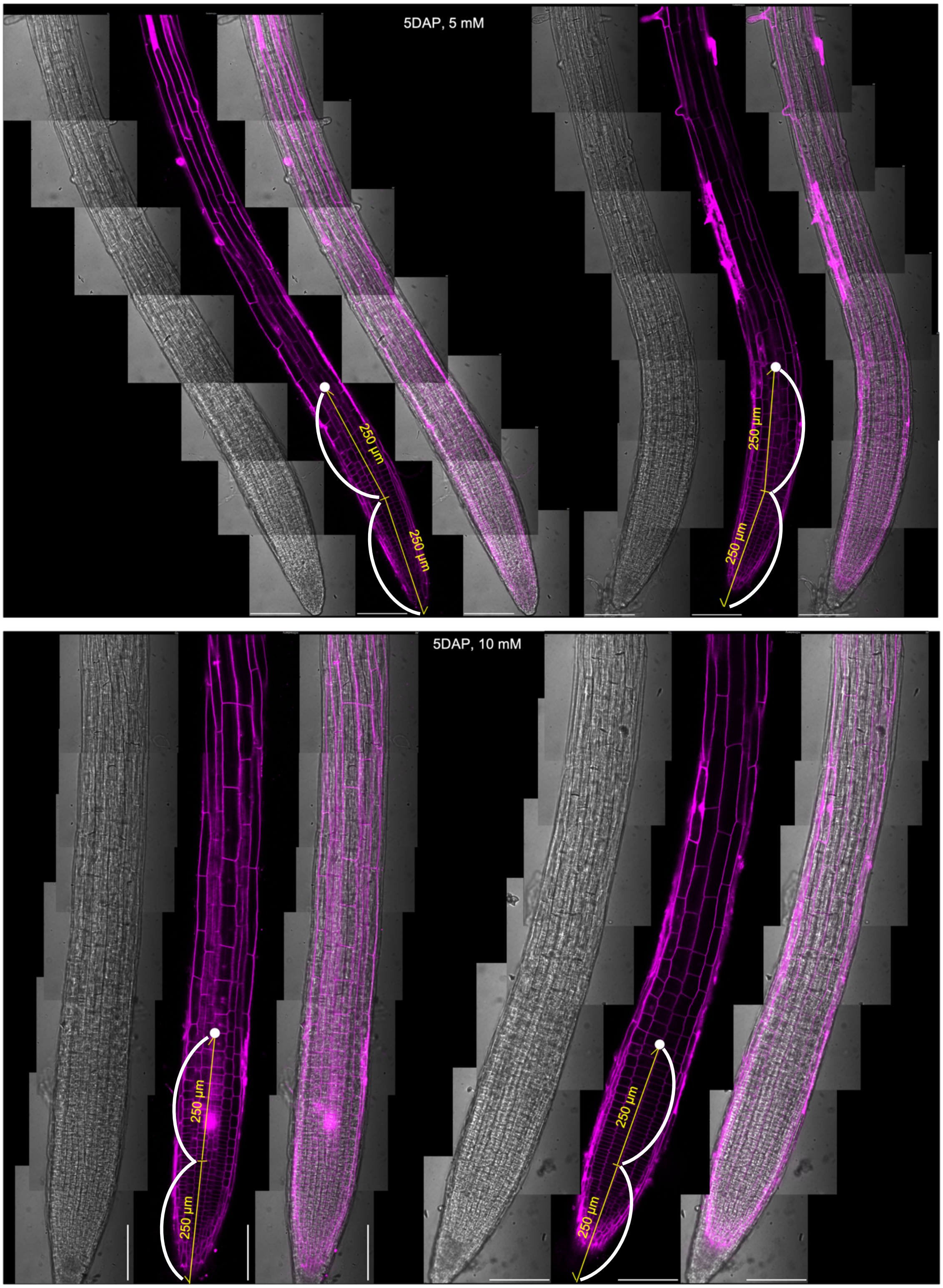

Supplementary Fig. S4. Additional 5 days after planting (DAP), PI stained-root images from 5 mM and 10 mM [KNO_3] plates. White dots indicate the starting point of clear root elongation. Root zones (indicated by arched white line) are marked in yellow text with 250 µm increments (in yellow line) measured from root tip. Scale bars indicate 100 µm.

Gene	ID	Zone 1	Zone 2	Zone 3	Zone 4
NPF6.3/NRT1.1	AT1G12110	2.80	1.18	6.73	16.22
NPF4.6/NRT1.2	AT1G69850	0.85	0.60	1.35	2.35
NRT2.1	AT1G08090	0.87	0.78	0.94	1.58
NRT2.2	AT1G08100	0.71	0.72	0.72	0.75
NRT2.4	AT5G60770	0.70	0.70	0.66	0.63
NRT2.5	AT1G12940	0.80	0.79	0.62	0.62
CLCa	AT5G40890	2.80	5.00	10.02	7.96
CLCb	AT3G27170	0.51	0.62	2.16	4.51

Supplementary Table S3. Average relative expression levels of nitrate transporter genes expressed in different developmental zones. The data has been obtained from Dinneny et al. 2008. The zone 1-4 roughly corresponds to 0-150, 150-350, 350-650, 650-1650 μm away from the root tip.
